# PupStruct: Prediction of Pupylated Lysine Residues Using Structural Properties of Amino Acids

**DOI:** 10.3390/genes11121431

**Published:** 2020-11-28

**Authors:** Vineet Singh, Alok Sharma, Abdollah Dehzangi, Tatushiko Tsunoda

**Affiliations:** 1 Faculty of Science Technology and Environment, University of the South Pacific, Suva, Fiji; 2 Institute for Integrated and Intelligent Systems, Griffith University, Brisbane, QLD 4111, Australia; 3 Laboratory for Medical Science Mathematics, RIKEN Center for Integrative Medical Sciences, Yokohama 230-0045, Japan; tsunoda@bs.s.u-tokyo.ac.jp; 4 School of Engineering and Physics, Faculty of Science Technology and Environment, University of the South Pacific, Suva, Fiji; 5 Department of Computer Science, Rutgers University, Camden, NJ 08102, USA; i.dehzangi@rutgers.edu; 6 Center for Computational and Integrative Biology, Rutgers University, Camden, NJ 08102, USA; 7 Laboratory for Medical Science Mathematics, Department of Biological Sciences, Graduate School of Science, The University of Tokyo, Tokyo 113-0033, Japan; 8 Department of Medical Science Mathematics, Medical Research Institute, Tokyo Medical and Dental University, Tokyo 113-8510, Japan

**Keywords:** post-translational modification (PTM), lysine pupylation, structural features, protein sequences, amino acids, prediction

## Abstract

Post-translational modification (PTM) is a critical biological reaction which adds to the diversification of the proteome. With numerous known modifications being studied, pupylation has gained focus in the scientific community due to its significant role in regulating biological processes. The traditional experimental practice to detect pupylation sites proved to be expensive and requires a lot of time and resources. Thus, there have been many computational predictors developed to challenge this issue. However, performance is still limited. In this study, we propose another computational method, named PupStruct, which uses the structural information of amino acids with a radial basis kernel function Support Vector Machine (SVM) to predict pupylated lysine residues. We compared PupStruct with three state-of-the-art predictors from the literature where PupStruct has validated a significant improvement in performance over them with statistical metrics such as sensitivity (0.9234), specificity (0.9359), accuracy (0.9296), precision (0.9349), and Mathew’s correlation coefficient (0.8616) on a benchmark dataset.

## 1. Introduction

Post-translational modifications (PTM) are referred to as changes of protein composition through the addition of small molecules to specific sites (amino acid residues) on the body of a protein. Those modifications are responsible for protein function regulation, cell functioning, subcellular localization, biological practices, and protein turnover in health and disease [1,2,3,4,5]. Different PTMs have been identified so far, including ubiquitination [6], methylation [7,8], acetylation [9], glycation [10], prolyl isomerization [11], succinylation [12,13,14,15,16], crotonylation [17], and phosphoglycerylation [18].

While there have been a lot of studies done on these PTMs, another modification named pupylation [19,20] has attracted much attention in the research community. The process whereby prokaryotic ubiquitin-like protein (Pup) [21,22] attaches to substrates for degradation via an isopeptide bond causing the modification of specific lysine residues is pupylation [23]. Although Pup and Ubiquitin (Ub) are similar, their amino acid sequence or structure are different. While Ub has 76 amino acids, Pup proteins are small, ranging from 60 to 70 residues in length [24]. Pupylation plays a key role in regulating various cellular procedures, such as signal transduction and protein degradation in prokaryotic cells [25,26]. The pupylation and ubiquitylation are the same in function [27], but the enzymology involved in ubiquitylation requires an activating enzyme, conjugating enzyme, and protein ligase. In contrast, pupylation only requires deamidase of Pup (DOP) [28] and proteasome accessory factor A (PafA) [19,24,29]. First, the C-terminal glutamine of Pup is deamidated to glutamate via DOP, then the deamidated Pup is attached to a specific lysine of substrate proteins by PafA. The tagging with Pup can render proteins as substrates for proteasomal degradation. The depupylation event in actinobacteria and the fact that some members harbor the pupylation gene locus without encoding proteasomal subunits proposes the assumption that pupylation might fulfill a larger role in regulation and cellular signaling [30]. Most prokaryotic pupylation remains unknown [31,32].

To understand the fundamentals of pupylation, it is critical to involve biological markers at the cellular level for detecting pupylation sites. Identifying a pupylated site through the traditional experimental process is demonstrated to be expensive, complex, inefficient, and time-consuming. To overcome these disadvantages, computational methods are more preferred and a prediction tool is needed.

There are a number of computational models developed with a different technique, but there are a lot of improvements that can be done for better performance. Some of these methods include the first proposed technique GPS-PUP, which employed a group-based prediction system (GPS) sequence encoding [33], and EnsemblePup [34] which incorporated the bi-profile Bayes feature extraction with support vector machine (SVM). Features such as position-specific scoring matrix (PSSM), secondary structure, amino acid index property (AAindex), conservation scores, and structural disorder score were employed with an SVM classifier to develop PrePup [35]. IMP-PUP [36] constructed features based on the composition of k-spaced amino acid pairs on a semi-supervised self-training SVM algorithm, while pbPUP [37] was developed with the profile-based composition of k-spaced amino acid pair (pbCKSAAP) encoding with the SVM classifier. PUL-PUP [38] made use of the SVM algorithm and positive-unlabeled learning with a composition of k-spaced amino acid pairs feature (CKSAAP), iPUP [39] also incorporated CKSAAP features. The structural, sequential, and evolutionary hallmarks features which included protein secondary structures, physicochemical properties, binary features, PSSM, and amino acid pairs and SVM classifier was employed to develop PupPred [40]. EPuL [41] incorporated only positive and unlabeled samples. The progress and challenges faced in protein pupylation sites prediction were discussed in [20]. CIPPN [42] was developed using a neural network and, most recently, PSSM-PUP [43] employed PSSM, which was converted into bigram probabilities for feature extraction with an LibSVM classifier was developed.

The benchmark datasets from the PupDB database [44] is used in most of the studies. While many of the methods used the composition of k-spaced amino acid pairs features, there are only three methods, namely PrePup [35], PUL-PUP [38], and PupPred [40], which involve secondary structural features. Despite several methods being presented so far, their performance in identifying pupylated lysine residues remains limited, and therefore better techniques are necessary to determine the pupylated and non-pupylated lysine residues correctly.

In this study, we propose a new predictor, named PupStruct, which utilizes structural features such as accessible surface area (ASA), secondary structure (helix, strand, and coil), and backbone torsion angles for predicting pupylated lysines. The peptide comprising 13 amino acids upstream and downstream of lysine residue was employed for feature extraction. The benchmark dataset PupDB database [44] consisting of 153 proteins was used with a high number of non-pupylated lysines over the pupylated lysines. To reduce data imbalance, we used a k-nearest neighbors cleaning treatment [45] and employed a support vector machine with a radial basis kernel function for pupylation prediction. Structural features that contribute to the better overall performance of PupStruct in comparison to other methods was used. Finally, PupStruct was compared with two benchmark predictors ([36] and [38]) which showed a significantly improved performance over them. PupStruct is able to predict pupylated lysines with 0.9234 sensitivity, 0.9359 specificity, 0.9296 accuracy, and 0. 8616 Mathew’s correlation coefficient.

## 2. Materials and Methods

We propose a computational method named PupStruct which employs nine structural features, including accessible surface area, secondary structure (helix, strand, and coil), and backbone torsion angles. The following sections discuss the benchmark dataset, different features, feature exaction for each lysine, and the support vector machine classifier used for pupylation site prediction.

### 2.1. Protein Dataset

As stated in the introduction, for this study, we have taken the protein sequences from PupDB databases [44]. It contained 153 protein sequences whose lysine residues are either pupylated or non-pupylated. We examined each protein sequence and retrieved whether it was composed of pupylation or non-pupylation residues. We attained 181 positive lysines (pupylated) and 2290 negative lysines (non-pupylated) which were used for this study. The next section explains various structural features computed from each of the protein sequences.

### 2.2. Structural Features

We retrieved each protein sequence and computed nine different features related to the accessible surface area, secondary structure, and backbone torsion angles. These features are also used in other existing predictors [12,18,42,46,47]. To achieve this, we employed the toolbox SPIDER2 [48,49] which has previously obtained good outcomes for prediction using accessible surface area [50,51,52,53], secondary structure [54,55,56,57], and backbone torsion angles [50,58,59,60,61]. SPIDER2 has also been used to extract the structural properties for other predictions [12,18,61,62,63,64]. The details for these structural properties are explained in the succeeding sections.

#### 2.2.1. Accessible Surface Area (ASA)

*ASA* refers to the accessible area of each amino acid to a solvent of the protein in 3D configuration [65,66,67]. Since the value of an amino acid involves the protein configuration, the predicted *ASA* value of individual amino acids displays vital information regarding the protein structure. We executed SPIDER2 on each protein sequence to compute an estimated numeric *ASA* value for each amino acid in the protein with known 3D structures [48]. It is wise to note that the predicted *ASA* value entirely depends on the sequence information which is mainly used by SPIDER2 for computation.

#### 2.2.2. Secondary Structure

**Figure 1:**
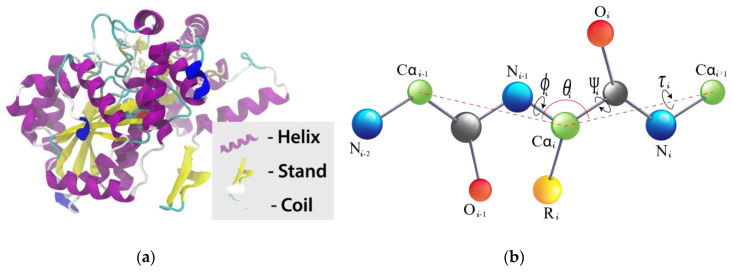
Illustrations of the secondary structure of a protein and local backbone torsion angles in amino acids (**a**) the helix, strand and coil for a protein (picture source: freepik.com) while (**b**) illustrates torsion angles associated with the protein backbone. Dihedral angle for the different bonds are discussed above.

This property presents significant information on the local 3D structure of proteins. This can be inferred as amino acid’s contribution to each of the defined local structures of proteins, namely helix (*ph*), strand (*pe*), and coil (*pc*), as is shown in Figure 1a. Again, we executed SPIDER2 to predict the prospect contribution of each amino acid to the three mentioned local structures, namely *ph, pe* and *pc*, which results in three discrete numerical vectors of these local structures [68]. Furthermore, SPIDER2 also gives the local structure with the highest probability as one *L* × 3 matrix, where *L* depicts the protein length, and the three columns are the corresponding probabilities contribution to each local structure *ph, pe* and *pc*. Hence, to simplify, we denote this matrix as *SSPre* [69].

#### 2.2.3. Local Backbone Torsion Angles

The secondary structure gives important information on local configuration of amino acids of protein [70], whereas torsion angles between neighboring amino acids supplement predicted *ASA* and secondary structure with vital information about the local structure of proteins. Since the predicted secondary structure is a distant output, the backbone torsion angle *ϕ* and *Ψ* continuously provides information on local amino acids interaction along the protein backbone [71,72]. Recently, two new angles are identified based on the dihedral angles *θ*, between three *Cα* atoms (*Cα_i-1_–Cα_i_–Cα_i+1_)* and τ, rotated about the *Cα_1_–Cα_i+1_* bond [50]. To attain these four angles, we executed SPIDER2 [49] on every protein sequence and achieved four numerical vectors, namely *ϕ, Ψ, θ* and *τ*. The illustration of *ϕ, Ψ, θ* and *τ* is shown in Figure 1b.

### 2.3. Feature Extraction for Lysine Residues

**Figure 2:**
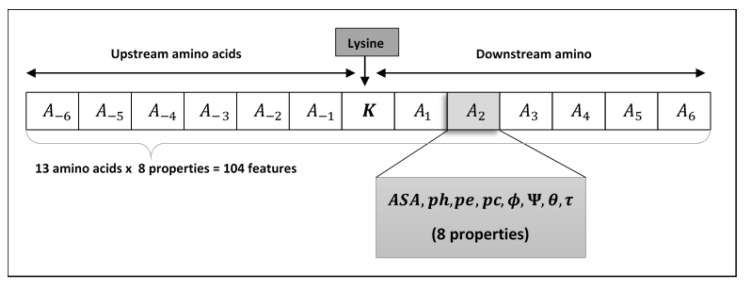
Shows arrangement of lysine residue’s neighboring amino acids with ample upstream and downstream amino acids.

**Figure 3:**
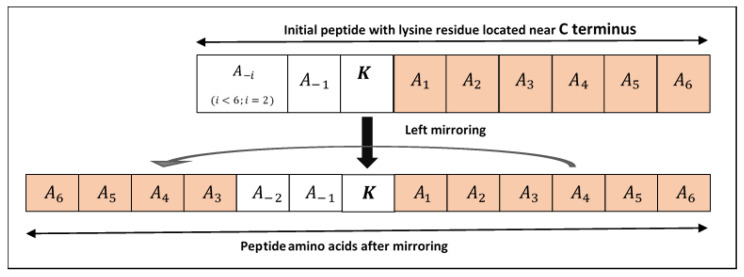
Shows lysine with insufficient amino acids. Left mirroring is illustrated to get sufficient upstream amino acids.

Structural features are used to identify the pupylated and non-pupylated sites by employing 6 upstream and 6 downstream amino acids from and including the lysine residue K as shown in Figure 2 which adds the window size equal to 13. The mirroring effect [18,47,73,74,75] was employed to fill the amino acids in the absence of 6 amino acids either upstream or downstream from the lysine residue, i.e., if the lysine residue is located near the *N* or *C* terminus as shown in Figure 3. To obtain the best window size, we constructed training dataset using 11- to 41-residue window sizes and trained the PupStruct predictor but the best result was obtained by window size 13.

Let us consider peptide S, consisting of 6 upstream and 6 downstream amino acids, including lysine reside K in the middle, that can be stated as:

(1) $$S=\;\{\;A_{-6},\;\;A_{-5},\;\;\;A_{-4},\;\;\;A_{-3},\;\;\;A_{-2},\;\;\;A_{-1},\;\;K,\;\;A_{1},\;\;A_{2},\;\;\;A_{3},\;\;\;A_{4},\;\;\;A_{5},\;\;\;A_{6}\text{\}}$$

Where *A-_i_* (for 1 ≤ *i* ≤ 6) are upstream and *A_i_* (for 1 ≤ *i* ≤ 6) are downstream amino acids. Therefore, the lysine residue consists of 13 amino acids in total, including *K*. Each peptide *S* will contain a pupylated or non-pupylated lysine which means the *K* can have a class label *x* as *x = {0, 1}* where *x = 1* then *S* denotes pupylated residue and if *x = 0* then *S* denotes non-pupylated residue. Moreover, each amino acid *A_i_* (for −6 ≤ *i* ≤ 6; *A_0_ = K*) can be deliberated by the structural features as:

(2) $$A_{i}=\left\{ASA,\;ph,\;pe,\;pc,\;\phi ,\Psi ,\theta ,\;\tau \right\}$$

It is worth noting that the structural features *ASA, ph, pe, pc, ϕ, Ψ, θ* and τ are numeric vales and each represents a sole value for each amino acid *A_i_*. Thus, *A_i_* can be expressed in an 8-dimensional feature vector. The numeric values were normalized and then placed in a vector form. This implies that each segment *S* (of 13 amino acids) is represented by 104 structural features (of 13 amino acids *x* 8). These structural features of lysine are used to predict pupylated or non-pupylated sites in line with the peptide *S*.

### 2.4. Support Vector Machine for Classification

The support vector machine (SVM) [76,77] is engaged in both regression and classification applications and is also used in many state-of-the-art predictors for pupylation sites [35,36,37,38,39,41,43]. The literature shows that the SVM produces a lower prediction error compared to other classifiers when large numbers of features are considered, as in this study there are 104 features. It is also proven that SVM has produced best results and mostly used in the areas of bioinformatics research including genomics, protein function prediction, protease functional site recognition, chemogenomics, transcription initiation site prediction and gene expression data classification [78,79,80]. SVM operates by discovering the maximum difference among the two hyperplanes demonstrating linear boundaries of two different classes. The dimension of the hyperplane is influenced by the number of features, therefore, kernel functions which can be either polynomial, radial or linear was employed to deal with non-linear boundaries between classes [81,82,83]. We used the LIBSVM [84] classifier developed Chih-Chung Chang and Chih-Jen Lin on the Matlab platform developed the MathWorks, Inc from Natick, Massachusetts, United States, with a radial basis kernel function to determine the margin between pupylated and non-pupylated lysine residues. The radial basis kernel function was fine-tuned with a gamma set to 0.5 and cost value set to 3 (nu-SVR).

## 3. Results

Having a computational method which aims to predict pupylation sites requires a severe assessment of its performance. This section discusses the statistical metrics, evaluation strategy, balancing dataset, oversampling dataset, and the comparison of the proposed PupStruct with other recent state-of-the-art predictors from the literature.

### 3.1. Performance Measures

In this study, we have incorporated five metrics to compare the performance of PupStruct with other state-of-the-art predictors in terms of predicting pupylated and non-pupylated lysines. These five metrics are sensitivity (Sn), specificity (Sp), accuracy (Acc), precision (Pre) and Matthew’s correlation coefficient (Mcc), which are widely used in the literature [35,37,38,39,85]

Sensitivity, which is one of the key measures, evaluates the correctness of identifying pupylation sites. The predictor attaining high sensitivity shows that it can accurately detect the pupylated lysines (positive instances). In other words, sensitivity value 0 shows the predictor’s inability to detect any pupylated lysine residues (true positives), whereas the value 1 depicts a predictor’s capability to correctly identify all pupylated lysines. Specificity gauges the predictor’s capability to detect non-pupylation sites (true negative). It varies between 0 and 1 where value 0 shows predictor’s inability to predict non-pupylation sites and value 1 indicates predictor’s ability to predict non-pupylation sites. Accuracy calculates the total number of correctly classified pupylated and non-pupylated lysine residues which ranges between 0 and 1 where 0 means a least accurate predictor and 1 means the best accurate predictor. Precision, which is another assessment measure, is a fraction of correctly identified pupylated sites over the sum of correctly identified pupylated and non-pupylated sites. Mathew’s correlation coefficient (MCC) scales the classification quality of the predictor, which ranges from −1 to +1. A predictor with (MCC) value −1 implies a totally negative correlation, whereas +1 means a completely positive correlation.

Considering Equations (3)–(7) for each metric, let’s look at a dataset with +P as a number of pupylated sites and −P as a number of non-pupylated sites. Therefore, each metric can be expressed as:

(3) $$Sensitivity=\frac{+P_{+}}{+P_{+}+\;+P_{-}}$$

(4) $$Specificity\;=\frac{-P}{-P_{+}+\;-P_{-}}$$

(5) $$Precision\;=\frac{+P_{+}\;+\;-P_{+}}{+P\;+\;-P}$$

(6) $$Accuracy\;=\frac{+P_{+}\;+\;-P_{+}}{+P\;+\;-P}$$

(7) $$MCC=\frac{\left(-P_{+}\;\times \;+P_{+}\right)-\left(-P_{-}\;\times \;+P_{-}\right)}{\sqrt{\left(+P_{+}\;+\;\;+P_{-}\right)\left(+P_{+\;}+\;\;-P_{-}\right)\left(-P_{-}\;+\;+P_{-}\right)\left(-P_{+\;}+\;\;-P_{-}\right)}}$$

where $+P_{+}$ is number of pupylated sites classified correctly, $+P_{-}$ represents the number of pupylated sites incorrectly classified, $-P_{+}$ is the number of non-pupylated sites predicted correctly and $-P_{-}$ represents the number of incorrectly predicted non-pupylated sites by the predictor.

The perfect predictor should achieve the highest in all the five metrics. However, at least sensitivity should be greater when comparing it with other predictors. A lower value of sensitivity shows that it cannot correctly predict pupylated lysine residues and, therefore, it is not fit for lysine pupylation detection.

### 3.2. Evaluation Strategy

To accurately evaluate the effectiveness of the PupStruct predictor in terms of the statistical metrics, we used a cross-validation method. Two most common cross-validation approaches are n-fold cross-validation and jackknife. An independent test set is used for evaluation purposes. The jackknife method is considered to be the least arbitrary and yields unique outcomes for a dataset [86] but we deployed the n-fold cross-validation scheme for this study which involves less processing time and also commonly used in the literature [15,35,36,38,39,41]. The n-fold cross-validation technique is employed in the following steps:

1. Partition data samples randomly into n parts of roughly equal size with roughly similar negative and positive samples on each fold.
2. Take out one-fold as test set or validation data and the remaining n-1 folds as training data.
3. Use the training data set to fine-tune the parameters of the predictor.
4. Use the test set to compute the five statistical metrics.
5. Repeat Step 1 to Step 4 for the remaining n folds and calculate the average of each performance metric.

We carried out 6-, 8- and 10-fold cross-validations to evaluate PupStruct predictor and recorded the result.

### 3.3. Filtering Out the Imbalance Data

Our benchmark dataset comprised 163 protein sequences which has 181 pupylation sites (positive sample set) and 2290 non-pupylation sites (negative sample set). The difference between the number of positive set and negative set of around 12 times creates a huge imbalance between the classes. Although this may be biologically realistic to have a number of non-pupylated lysines greater than pupylated lysines, this inconsistency can cause severe bias in machine learning. We applied the k-nearest neighbors cleaning technique [45] to tackle this problem, which is mostly used in the literature.

For this, we first set the cut-off K value equal to 12 since the negative and positive sample ratio was about 12:1, thus we eliminated any negative sample which had at least one positive sample within its 12-nearest neighbors. We consequently increased the k value until we achieved similar numbers of positive and negative samples. Eventually, the number of negative samples was significantly reduced to 188 samples by k value of 48. After the filtering process, the filtered negative samples and all positive samples were used to perform n-fold cross-validation to evaluate the PupStruct predictor.

### 3.4. PupStruct vs. Other Existing Predictors

The proposed PupStruct was compared with recent two proposed predictors IMP-PUP [36] and PUL-PUP [38]. It can be noted that PUL-PUP also used structural features. The software packages were given for the two methods. It is worth noting that both predictors used the same dataset thus, many of the proteins may be used in their training set. Therefore, the software was re-run and tested using the test set respectively to the set used in PupStruct evaluation process. For PUL-PUP [38], since the code didn’t execute, we retrieve the features from the method and used the same train and test set from PupStruct to calculate the performance. The performance reported is based on test data which correspond to the test set kept aside during the n-fold cross-validation procedure means that we keep aside the test set during the n-fold cross-validation procedure. Test data was not used to adjust the training parameters of the model.

Table 1 reports the performance of the predictors. It is clearly witnessed that the proposed PupStruct is performing better than all the benchmark predictors in metrics in likes of sensitivity, accuracy and MCC. The sensitivity was improved by 14%, accuracy by 11%, specificity by 7%, and precision by 9%. Moreover, MCC was significantly improved by 21% compared to IMP-PUP [36].

**Figure 4:**
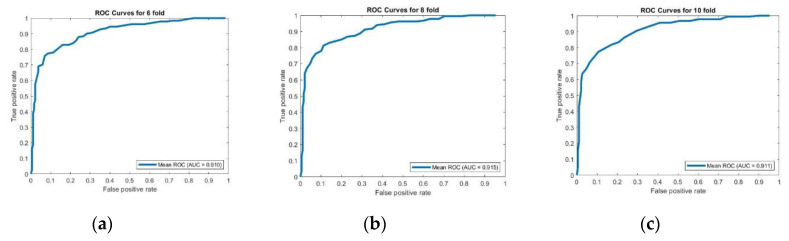
Shows mean Receiver operating characteristic (ROC) curves of PupStruct predictions on 6-, 8- and 10-folds (**a**) ROC curves for 6 folds, (**b**) ROC curves for 8 folds, (**c**) ROC curves for 10 folds. This encouraging result demonstrates the proficiency of proposed PupStruct predictor to distinguish the pupylated and non-pupylated lysine residues accurately. It appears that structural information of amino acids provides essential information about nearby modified lysines. Finally, the SVM classifier with a radial basis kernel function appears to discover the maximal separation of both hyperplanes when structural characteristics are employed. All these structural features together with the classifier plays a key role in predicting pupylated and non-pupylated lysine residues.

To give more insight of the performance of PupStruct, we generated ROC curve to measure AUC (area under the curve) and calculated the average AUC values for 6-, 8-, and 10-fold cross validations which was recorded at 0.910, 0.915 and 0.911 respectively which indicates stable performance of PupStruct. The results of the ROC-AUC analysis are shown in Figure 4.

Our PupStruct predictor’s software package can be accessed from https://github.com/vinzsingh09/PupStruct.

## 4. Discussion

We further analyzed each feature (accessible surface area, secondary structure (helix, strand, and coil), and backbone torsion angles) to gauge their contribution towards the predictor. We used different features to train and test the model and recorded the result for comparison. Initially, we used each group of features for training, that is ASA then secondary structure (helix, strand, and coil) and backbone torsion angles. Next, we used individual features (ASA, ph, pe, pc, ϕ,Ψ,θ, τ) separately and recorded their result contributing to the predictor. Finally, we used a combination of some of these features which are contributing the most towards the predictor. The result shown in Table 2 is for six-fold cross validation.

It is clearly observed from Table 2 that *ASA* and secondary structure (*ph*, *pe*, *pc,* also known as *SSpre*) contributes the most towards the performance. It observed that *SSPre* has contributed the most towards specificity and precision, while *ASA* contributes the most towards sensitivity and MCC, which are the most important metrics. However, combining the two reduces the performance. Protein’s shape is determined by amino acid sequence in the polypeptide chain. When exposed to the cytosol (water-based solution in which proteins floats) or lumen (inside space of a tubular structure), polypeptide chain assumed to localized organization to secondary structure that optimizes interactions between side chains of amino acids with each other and water. The polypeptide backbone folds into spirals (helix) and ribbons (stand). These properties provide very important information about the amino acid and extracting the helix, stand, and coil (*SSPre*) values contributed the most to the performance of PupStruct [87,88]. In the literature, the accessible surface area (*ASA*) of a protein is always considered as a determining factor in protein folding and stability work. *ASA* is surface characterized around a protein by a hypothetical centre of a solvent sphere with the surface of the molecule. Based on the *ASA* value, amino acid residues can be determined as buried or exposed. This makes *ASA* a crucial feature contributing towards the performance [88]. When considering the individual feature only, then *ASA*, coil (*pc*), followed by Tau from the local torsion angle contributes the most towards the predictor. Eventually, using all the features gave the best result, which is shown in Table 1, which means that each feature demonstrated some contribution towards the predictor.

## 5. Conclusions

This study presented a new computational method named PupStruct for identifying pupylation sites in protein sequences. PupStruct utilizes structural information of amino acids around the lysine residue and uses the k-nearest neighbour approach to solve the imbalance data issue. The analysis of which features contribute how much to the predictor was crucial information for training. Finally, the support vector machine (LIBSVM) with a radial basis kernel function to identify maximal separation between pupylated and non-pupylated lysine residue showed that PupStruct performed better than the existing predictors.

## Figures and Tables

**Table 1:** Shows comparison of performance assessment of PupStruct and two benchmark predictors for 6-, 8-, 10-fold cross-validation. The highest values in each metric are in bold.

Fold	Predictor	Sensitivity	Specificity	Precision	Accuracy	MCC
6	PUL-PUP	0.5586	0.7547	0.6897	0.6586	0.3219
	IMP-PUP	0.7785	0.8611	0.8407	0.8205	0.6437
	PupStruct	0.9228	0.9309	0.9317	0.9270	0.8563
8	PUL-PUP	0.5753	0.7919	0.7308	0.6856	0.3826
	IMP-PUP	0.7767	0.8610	0.8422	0.8197	0.6423
	**PupStruct**	**0.9234**	**0.9359**	**0.9349**	**0.9296**	**0.8616**
10	PUL-PUP	0.6082	0.7190	0.6946	0.6646	0.3380
	IMP-PUP	0.7784	0.8611	0.8429	0.8203	0.6441
	PupStruct	0.9173	0.9409	0.9398	0.9296	0.8611

**Table 2:** Shows features and what percentage it contributed towards the predictor.

Feature	Sn (%)	Sp (%)	Pre (%)	Acc (%)	MCC (%)
*ASA*	86.70251	87.83602	87.60489	87.26766	0.74812
*Ph, Pe, Pc (SSPre)*	81.75627	92.89773	91.29129	87.56934	0.754546
Helix (*Ph*)	65.08961	93.59879	90.65543	79.61739	0.615526
Strand (*Pe*)	43.15412	95.39141	91.2274	70.2561	0.461857
Coil (*Pc*)	81.72043	89.78495	89.19853	85.81879	0.723357
Local Torsion angle	75.71685	69.80843	71.79877	72.77045	0.457679
Phi	76.73835	78.88889	78.17483	77.80965	0.560354
Psi	75.66308	76.88172	76.33012	76.26917	0.526891
Theta	61.21864	81.49425	77.14761	71.3354	0.438473
Tau	80.10753	81.1828	80.70276	80.64075	0.615214
*ASA + Sspre*	77.921147	88.25605	86.53159	83.18623	0.66673
